# Identifying molecular and functional similarities and differences between human primary cardiac valve interstitial cells and ventricular fibroblasts

**DOI:** 10.3389/fbioe.2023.1102487

**Published:** 2023-03-27

**Authors:** Martha E. Floy, Fathima Shabnam, Sophie E. Givens, Vaidehi A. Patil, Yunfeng Ding, Grace Li, Sushmita Roy, Amish N. Raval, Eric G. Schmuck, Kristyn S. Masters, Brenda M. Ogle, Sean P. Palecek

**Affiliations:** ^1^ Department of Chemical and Biological Engineering, University of Wisconsin-Madison, Madison, WI, United States; ^2^ Department of Biomedical Engineering, University of Minnesota, Minneapolis, MN, United States; ^3^ Department of Biomedical Engineering, University of Wisconsin-Madison, Madison, WI, United States; ^4^ Department of Chemical Engineering, University of Florida, Gainesville, FL, United States; ^5^ Department of Medicine, University of Wisconsin-Madison, Madison, WI, United States; ^6^ Stem Cell Institute, University of Minnesota, Minneapolis, MN, United States

**Keywords:** cardiac, fibroblast, heart, valve, ventricle, comparison, human

## Abstract

**Introduction:** Fibroblasts are mesenchymal cells that predominantly produce and maintain the extracellular matrix (ECM) and are critical mediators of injury response. In the heart, valve interstitial cells (VICs) are a population of fibroblasts responsible for maintaining the structure and function of heart valves. These cells are regionally distinct from myocardial fibroblasts, including left ventricular cardiac fibroblasts (LVCFBs), which are located in the myocardium in close vicinity to cardiomyocytes. Here, we hypothesize these subpopulations of fibroblasts are transcriptionally and functionally distinct.

**Methods:** To compare these fibroblast subtypes, we collected patient-matched samples of human primary VICs and LVCFBs and performed bulk RNA sequencing, extracellular matrix profiling, and functional contraction and calcification assays.

**Results:** Here, we identified combined expression of *SUSD2* on a protein-level, and *MEOX2*, *EBF2* and *RHOU* at a transcript-level to be differentially expressed in VICs compared to LVCFBs and demonstrated that expression of these genes can be used to distinguish between the two subpopulations. We found both VICs and LVCFBs expressed similar activation and contraction potential *in vitro*, but VICs showed an increase in ALP activity when activated and higher expression in matricellular proteins, including cartilage oligomeric protein and alpha 2-Heremans-Schmid glycoprotein, both of which are reported to be linked to calcification, compared to LVCFBs.

**Conclusion:** These comparative transcriptomic, proteomic, and functional studies shed novel insight into the similarities and differences between valve interstitial cells and left ventricular cardiac fibroblasts and will aid in understanding region-specific cardiac pathologies, distinguishing between primary subpopulations of fibroblasts, and generating region-specific stem-cell derived cardiac fibroblasts.

## 1 Introduction

Cardiac fibroblasts (CFBs) play a key role in cardiovascular diseases by contributing to inflammation and fibrotic scarring, and therefore are a potential therapeutic target. Many clinical cardiac pathologies occur in specific regions of the heart; for example, the majority of arrythmias occur in the atria (Sánchez et al., 2019), fibrosis mainly occurs in the left ventricle (Kanisicak et al., 2016), and mineralization occurs primarily in the heart valves and vasculature (Li et al., 2021). Hence, heterogeneity in CFBs from different regions of the heart likely contributes to regional clinical pathologies which may arise by patterning through developmental lineage progression and local environmental cues (Tallquist, 2020). However, it has been difficult to distinguish between lineage specification and local environmental specification using *in vivo* models.

In this study, we identify molecular and functional similarities and differences between valve interstitial cells (VICs) and left ventricular cardiac fibroblasts (LVCFBs). VICs are the interstitial cells located in all three layers of the heart valve (fibrosa, spongiosa, and ventricularis/atrialis), the surface of which is lined by valvular endothelial cells (Menon and Lincoln, 2018). This heterogenous CFB population is thought to play a key role in regulating ECM deposition of collagen, proteoglycans, and elastin during valve development and homeostasis (Aikawa et al., 2006; Liu et al., 2007). LVCFBs are located in the myocardium of the left ventricle and provide key support to endothelial cells and cardiomyocytes, heart muscle cells (Moore-Morris et al., 2014; Nicin et al., 2022). Lineage tracing studies using mouse Cre-based genetic tracing strategies have shown that VICs arise from both epicardial and endocardial cell progenitors with the composition varying between valves, with the majority of cells in the parietal and mural leaflets of the atrioventricular valves originating from epicardial cells and the majority of septal and aortic leaflets originating from endocardial cells (Wessels et al., 2012). In comparison, CFBs located in the heart wall are thought to be almost entirely derived from epicardial lineages (Moore-Morris et al., 2014). Despite a similar progenitor lineage, we hypothesize that LVCFBs and VICs have distinct transcriptional and functional differences that guide their specific roles in myocardium and valve tissues, respectively.

Here, we report molecular and functional differences *in vitro* between VICs and LVCFBs. This work identifies key similarities such as contraction potential, and differences such as increased calcification potential in LVCFBs compared to VICs. Furthermore, we analyzed differentially regulated genes and proteins between the two populations and identified marker genes and pathways as well as ECM proteins differentially regulated between VICs and LVCFBs. Characterizing the molecular and functional differences between these cells adds to our ability to distinguish between these subpopulations and increases understanding of the function of these region-specific populations. For example, while calcification is typically observed in valves in clinical pathologies, our *in vitro* experiments indicate under similar culture conditions, LVCFBs exhibit higher calcification compared to VICs. Furthermore, while collagens and matricellular proteins such as periostin have been studied in VICs, we show that LVCFBs secrete a higher abundance of these proteins. Instead, clotting ECM proteins and some matricellular proteins such as cartilage oligomeric matrix protein and alpha-2-HS-glycoprotein were secreted at a higher abundance in VICs. The comparative findings in this paper may also be used for generating and identifying region-specific stem cell-derived cardiac fibroblasts to advance *in vitro* modeling of cardiac tissue and cell-based cardiac regenerative therapies.

## 2 Materials and methods

### 2.1 Donor approval

Human tissues were procured and used with local Institutional Review Board approval. Donor hearts (*n* = 3, 1 male, 2 female, ages 39, 33, and 34 years old respectively) were obtained from the University of Wisconsin Organ Procurement Organization, Madison, WI from brain dead patients undergoing routine organ and tissue donation for transplant. These hearts were considered healthy, but were unused for transplant. Hearts were harvested by aseptical excision followed by perfusion and storage in cold cardioplegia solution prior to transport on ice. Hearts were processed between 4–6 h post explant.

### 2.2 Primary cell isolation

To isolate left ventricular cardiac fibroblasts, epicardial fat was removed and 25–30 g of left ventricular free wall was minced and transferred into gentleMACS C tubes (Miltenyi Biotec). Then, 10 mL digestion medium (DMEM supplemented with 0.125 mg/mL Liberase TM (Roche)) was added to each tube. Samples were homogenized using gentleMACS Dissociator (Miltenyi Biotec) using a C-tube, and incubated at 37°C for 30 min under agitation. The process was repeated twice to ensure tissue dissociation. Heart samples were then run through a 200 μm filter and centrifuged at 1000 *g* for 20 min. The supernatant was aspirated, and the remaining cell pellet was suspended in 20 mL primary CFB maintenance medium (MCDB131 basal medium (Gibco) supplemented with 10% FBS (R&D Systems), 5 μg/mL insulin (Sigma-Aldrich), 1 ng/mL bFGF (R&D Systems), 0.01 mg/mL ciprofloxacin, and 2.5 μg/mL Amphotericin B) (Gibco) and plated into T75 flasks. 2 h later, non-adherent cells were removed by washing in DPBS. Finally, primary CFB maintenance medium was replaced, and cells were cultured at 37°C, 5% CO_2_, and 100% humidity.

To isolate valve interstitial cells, aortic valve leaflets were excised and washed in PBS (no Ca^2+^, no Mg^2+^). They were then incubated in a 2.5 mg/mL collagenase type II solution (Worthington) for 1 h. After incubation, valve leaflets were vortexed at the highest setting for 1 min to remove endothelial cells. The tissue was then further digested in a 1 mg/mL collagenase type II solution for 3 h and vortexed again for 2 min followed by pipetting with a 25 mL disposable pipette to release the valve interstitial cells. The cell suspension was centrifuged, and the resulting cell pellet was resuspended and plated.

### 2.3 Primary cell maintenance

Primary CFBs and VICs were maintained in primary CFB maintenance medium (MCDB131 basal medium supplemented with 10% FBS, 5 μg/mL insulin, 1 ng/mL bFGF, 0.01 mg/mL ciprofloxacin, and 2.5 μg/mL Amphotericin B) with medium changes every 2 days. These culture conditions have been demonstrated to maintain the native phenotype of healthy VICs (Porras et al., 2017). At 80% confluence, primary CFBs and VICs were passaged for up to six passages using Accutase or 1X TrypLE for 10 mins in the incubator at 37°C, 5% CO_2_, and 100% humidity or until cells were singularized and then quenched with maintenance media, and centrifuged for 5 mins at 200 g.

### 2.4 Flow cytometry

Cells were singularized and fixed in 1% PFA for 15 min and stored in 90 (v/v)% methanol in water at −20°C until staining. Cells were then washed with 0.5% BSA in PBS (no Ca^2+^, no Mg^2+^) and stained with 100 μL of primary antibodies resuspended in 0.5% BSA with 0.1% Triton-X100 in PBS (no Ca^2+^, no Mg^2+^) in the ratio given in Supplementary Table S2. Samples were then incubated overnight at 4°C on a shaker. The following day, samples were washed with 0.5% BSA in PBS (no Ca^2+^, no Mg^2+^) and stained with 100 μL of secondary antibodies resuspended in 0.5% BSA with 0.1% Triton-X100 in PBS (no Ca^2+^, no Mg^2+^) at 1:1,000 according to Supplementary Table S2. Samples were then incubated for 30 min at room temperature, washed with 0.5% BSA in PBS (no Ca^2+^, no Mg^2+^), and analyzed on a BD Accuri C6 Plus cytometer. At least 10,000 events were collected per sample and analysis was performed using FlowJo software.

### 2.5 Immunocytochemistry and cell area analysis

Cells were fixed in 4% PFA for 15 min, and blocked using 0.5% BSA with 0.1% Triton-X100 in PBS (no Ca^2+^, no Mg^2+^) or 0.5% dry milk with 0.4% Triton-X100 in PBS (no Ca^2+^, no Mg^2+^) for 1 h at room temperature or overnight at 4°C. Then, cells were treated with primary antibody solution (see Supplementary Table S2) resuspended in 0.5% BSA with 0.1% Triton-X100 in PBS (no Ca^2+^, no Mg^2+^) or 0.5% dry milk with 0.4% Triton-X100 in PBS (no Ca^2+^, no Mg^2+^) and incubated overnight at 4°C. The following day, samples were washed with PBS (no Ca^2+^, no Mg^2+^), treated with secondary antibody solution at 1:1,000 (see Supplementary Table S2, and incubated for 1 h at room temperature or overnight at 4°C. Finally, samples were washed with PBS (no Ca^2+^, no Mg^2+^), counterstained with 5 μg/mL Hoechst, and imaged using a Nikon Ti-2 inverted microscope. Image analysis was performed using ImageJ Fiji software to automatically threshold and tabulate cell attributes.

### 2.6 mRNA extractions

Cells were singularized in Accutase, quenched, and centrifuged for 5 min at 200 g. Cell pellets were snap frozen at −80°C until further extraction. Total mRNA was isolated using the Qiagen RNeasy mini kit and treated with DNase (Qiagen). mRNA was eluted using DNA/RNA free water and stored at −80°C until sequencing.

### 2.7 cDNA preparation and RT-qPCR analysis

Using the Ominiscript Reverse Transcriptase kit (Qiagen) and Oligo (dT)_20_ Primers, 500 ng mRNA was reversed transcribed into cDNA. Real-time quantitative PCR with two technical replicates in 25 μL reactions using PowerUP Syber Master Mix were analyzed on an AriaMx Real-Time PCR System with an annealing temperature of 60°C. *RPL13A* was used as the housekeeper and analysis was performed using the ΔCt method. Primer sequences can be found in Supplementary Table S3.

### 2.8 Sequencing sample preparation and processing

Quantity and quality of RNA samples were first analyzed for the presence of 18 S and 28 S ribosomal RNA with A_260_/A_280_ ratio around 2.0 and A_260_/A_230_ ratios greater than 2.0. RNA was next quantified on an Agilent 2,100 Bioanalyzer using Qubit prior to library construction and sequencing. Sequencing libraries were constructed using the Illumina TruSeq Stranded mRNA kit and sequenced on an Illumina NovaSeq6000. Between 10 and 25 million reads were uniquely mapped per sample (Supplementary Material S2).

Raw FASTQ files were mapped to the human genome (hg38 + decoy) using STAR v2.5.3a (Dobin et al., 2013). Gene-level transcript abundances were calculated using featureCounts v2.0.3 (Liao et al., 2014). DESeq2 and all following commands were performed using R v4.1.3 (Love et al., 2014). DESeq2 was performed with a multi-factor design to account for the paired donor-matched samples. PCA plot was performed on variance-stabilized counts values, and differential gene lists were obtained with a threshold of p-adj<0.05. We also performed QuaternaryProd analysis using package v1.28.0 and gene ontology (GO) analysis using PANTHER with Reactome pathway database (Ashburner et al., 2000; Fakhry et al., 2016; Gene Ontology Consortium et al., 2021). Processed data including differential gene lists, transcripts per million (TPM) list, QuartneryProd and GO analysis are available in Supplementary Material S2.

We obtained count matrices for publicly available single cell sequencing datasets (GSE194180 and SCP498, Broad Institute) and selected the desired cells. UMAP and dot plots were prepared by the authors using the Seurat package version 4 (Hao et al., 2021).

### 2.9 Western blot analysis

Cell lysates were collected by treating cells with RIPA buffer in the presence of Halt Protease Inhibitor Cocktail (ThermoFisher). A BCA assay was used to determine the total protein concentration of cell lysates. Equal mass of protein was loaded on a 4%–12% tris-glycine gel under reducing conditions and transferred to nitrocellulose membranes. Membranes were blocked in tris-buffered saline +0.1% Tween20 (TBST) + 5 (wt/vol)% milk buffer for 1 h at room temperature. 15 mL of primary antibody solution (see Supplementary Table S2) were added to each membrane and incubated overnight at 4°C on a shaker. The following day, membranes were washed with TBST, incubated with secondary antibodies at 1:5,000 (see Supplementary Table S3) for 1 h at room temperature on a shaker, and washed again in TBST. Blots were then imaged on an LICOR Odyssey system, and bands were quantified using Image Studio 5.2.

### 2.10 Collagen gel contraction

Collagen gel contraction assays were performed with three wells per primary CFB line with a seeding density of 5 × 10^6^ cells/mL in primary CFB maintenance medium according to manufacturer’s instructions for the Cell Contraction Assay kit (CBA5020, Cell Biolabs). The gels were imaged after 24 h and gel area was quantified using ImageJ Fiji software.

### 2.11 Osteogenic induction

Cells were plated at 7,000 cells/cm^2^ in a 6-well plate and cultured in primary CFB maintenance medium or αMEM (Gibco) with 10% FBS, 50 mg/L L-ascorbic acid 2-phosphate sesquimagnesium salt hydrate (Sigma-Aldrich), 10 mM β-glycerophosphate disodium salt hydrate (Chem Impex), 10 nM dexamethasone (Sigma-Aldrich), 0.01 mg/mL ciprofloxacin, and 2.5 μg/mL Amphotericin B) as has previously been used for osteoinduction (Masjedi et al., 2016; Pillai et al., 2017; Kostina et al., 2018). Medium was changed every 2 days for 4 weeks.

### 2.12 ALP activity assay

Lysate samples were normalized by total protein content using the BCA assay. Equal amounts of protein were used in the alkaline phosphatase diethanolamine activity kit (Sigma-Aldrich). Each well contained 190 μL reaction buffer, 0.4 μL p-nitrophenyl phosphate, and 10.5 μL sample lysate. Two technical replicates per sample were averaged and absorbance (405 nm) was measured on a Tecan M100 plate reader every 15 min while within the detection range of the plate reader.

### 2.13 Alizarin red staining and quantification

Cells were fixed in 4% paraformaldehyde for 15 min and washed with deionized water. Samples were stained with 0.5 mL/well (in a 6-well plate) of 40 mM Alizarin red for 30 min at room temperature on a rotary shaker. Samples were next washed four times with 1 mL/well DI water to remove excess stain, all liquid was removed from the wells, and they were imaged on an EVOS XL Core Imaging System. To quantify Alizarin red staining, samples were treated with 600 μL/well 10 (wt/vol)% cetylpyridinium chloride in 10 mM sodium phosphate at room temperature on a shaker for 1 h. Then, sample was diluted at 1:10 using deionized water, 150 μL was transferred into two wells of a 96-well plate (to account for variability in absorbance readings) and absorbance (560 nm) was measured on a Tecan M100 plate reader.

### 2.14 Von Kossa staining

Following Alizarin red staining and quantification, cells were washed with deionized water. Then, they were stained using a von Kossa staining kit (Abcam). Briefly, wells were incubated in 0.5 mL/well silver nitrate solution (5%) for 20 min with exposure to ultraviolet light. Wells were then washed three times with 2 mL/well deionized water. Cells were next incubated with 0.5 mL/well sodium thiosulfate solution (5%) for 2 min and washed three times with 2 mL/well deionized water. Lastly, cells were incubated with 0.5 mL/well Nuclear Fast Red solution for 5 min and washed with 2 mL/well deionized water three times prior to imaging on an EVOS XL Core Imaging System.

### 2.15 High-density culture and decellularization

High density fibroblast culture and decellularization protocols were performed as described previously (Floy et al., 2021). Fibroblasts were seeded at 7,000 cells/cm^2^ in a 48-well plate and cultured for 16 days without passaging. At day 16, cells were washed with PBS (no Ca^2+^, no Mg^2+^) followed by washes using wash buffer 1 (100 mM Na_2_HPO_4_, 2 mM MgCl_2_, 2 mM EDTA). Cells were then lysed in lysis buffer (8 mM Na_2_HPO_4_, 1% Triton X-100) and incubated at 37°C for 1 h. Following lysis, matrices were washed with wash buffer 2 (100 mM Na_2_HPO_4_, 300 mM KCl) and DI water. Plates were allowed to dry overnight in a biosafety cabinet and stored at −20°C prior to further sample processing.

### 2.16 High-density culture mass spectrometry sample preparation

Prior to trypsination, plates were removed from −20°C and allowed to reach room temperature for 20 min. Decellularized protein was dissolved in 75 μL of 6 M urea with 2.75 μL of 200 mM dithiothreitol (DTT) and incubated for 1 h at room temperature. Then, 15 μL of 200 mM iodoacetamide was added to each well, contents were thoroughly mixed, and samples were incubated for 1 h at room temperature in the dark. An additional 15 μL of DTT was added to each well, contents were thoroughly mixed, and samples were incubated for 1 h at room temperature in the dark. Samples were then quenched with 340 μL of 1 mM calcium chloride and for the optimal trypsin activity sample pH was adjusted to 7.8–8.7 with sodium hydroxide. 5 μL of 1 μg/μL Trypisn Gold, Mass Spectrometry Grade (Promega) was added to the wells, and samples were incubated for 24 h at 37°C. The next day, the peptide solution was transferred from the well plate to Eppendorf LoBind microcentrifuge tubes, frozen at −80°C for at least 3 h, and lyophilized overnight.

Protein purification was performed using the ZipTip C18 protocol. Lyophilized samples were reconstituted in reconstitution solution (5:95 Acetonitrile [ACN]:H2O, 0.1% trifluoroacetic acid [TFA]) and pH was adjusted to be less than 3 using 10% TFA. ZipTipsC18 were hydrated by aspirating and expelling hydration solution (50:50 ACN:H_2_O, 0.1% TFA) from the ZipTipC18 twice followed by hydration using the wash solution twice (0.1% TFA in H2O). Samples were loaded into the ZipTipsC18 by aspirating and expelling the reconstituted sample from the ZipTipC18 six times. Then, samples were desalted by washing three times with wash solution (0.1% TFA in water). Purified proteins were eluted into an Eppendorf LoBind microcentrifuge tube containing elution solution (60:40 ACN:H_2_O, 0.1% TFA). Eluted samples were frozen, lyophilized, and stored at −80°C prior to further analysis.

### 2.17 Mass spectrometry data acquisition, processing, and analysis

Decellularized high-density fibroblast purified proteins were reconstituted and analyzed using 1D capillary mass spectrometry on the Thermo Orbitrap Velos. Raw mass spectrometry data were processed using Proteome Discoverer 2.5 Software human UniProt dabatase. Any proteins detected from cellular debris were excluded, and proteins with a sum of posterior error probability (PEP) score less than 2 were excluded to minimize false positive protein detection. Label-free quantification methods were used to determine the relative abundance of individual ECM proteins as well as the abundance ratio of each ECM protein using VICs as the numerator and LVCFBs as the denominator. A *p*-value for each abundance ratio was calculated to determine if the protein is either present in increased or decreased abundance in the VIC samples as compared to the LVCFBs.

### 2.18 Statistics and experimental replication

For primary cell experiments, three donors were identified (ages 33, 34 and 39) and left ventricular cardiac fibroblasts and VICs were isolated from the same hearts. Unless otherwise noted, experiments contained at least three technical replicates. Mean and standard deviation bars are plotted in gray in the figures. Statistics were performed in JMP Pro V15 using a paired Student’s *t*-test, ANOVA, or ANOVA with Tukey’s *post hoc* test.

## 3 Results

### 3.1 Molecular characterization of primary CFBs reveals distinct CFB signatures

To investigate differences in fibroblast marker expression between VICs and LVCFBs, we isolated paired samples from 3 donors (Supplementary Table S1). We then verified expression of CFB surface and internal protein markers by flow cytometry and immunofluorescence staining. Flow cytometry results showed similar expression of CD90, fibronectin (FN), vimentin (VIM), and TE7 in both primary CFB populations (Supplementary Figure S1). Immunofluorescence staining also showed similar overall expression of fibroblast specific protein 1 (FSP1), TE7, VIM, collagen type 1 alpha 1 chain (COL1A1, and FN in both populations (Supplementary Figure S2). SUSD2 has been previously identified as enriched in left ventricular cardiac fibroblasts compared to dermal fibroblasts (Schmuck et al., 2021). Interestingly, SUSD2 protein expression was only observed in the LVCFBs while minimal expression was observed in the VICs by Western blotting, suggesting that these two populations have unique molecular signatures and SUSD2 can be used for distinguishing between the two CFB populations (Supplementary Figure S3).

*In vitro,* these two CFB populations had similar growth rates when cultured on uncoated tissue culture plastic in a low glucose maintenance medium (Supplementary Figure S4). To assess differences in morphology, we stained CFB filamentous actin with phalloidin and performed blinded image analysis on four well replicates per donor. We observed a larger cell area, perimeter, and increased circularity in the VICs compared to the LVCFBs (Supplementary Figure S4B–E). However, we observed a larger median forward scatter area in the LVCFBs compared to the VICs (Supplementary Figure S1) suggesting that the VICs exhibit a greater degree of spreading when plated than LVCFBs.

### 3.2 Bulk transcriptomics reveal differential genes between LVCFBs and VICs

**FIGURE 1:**
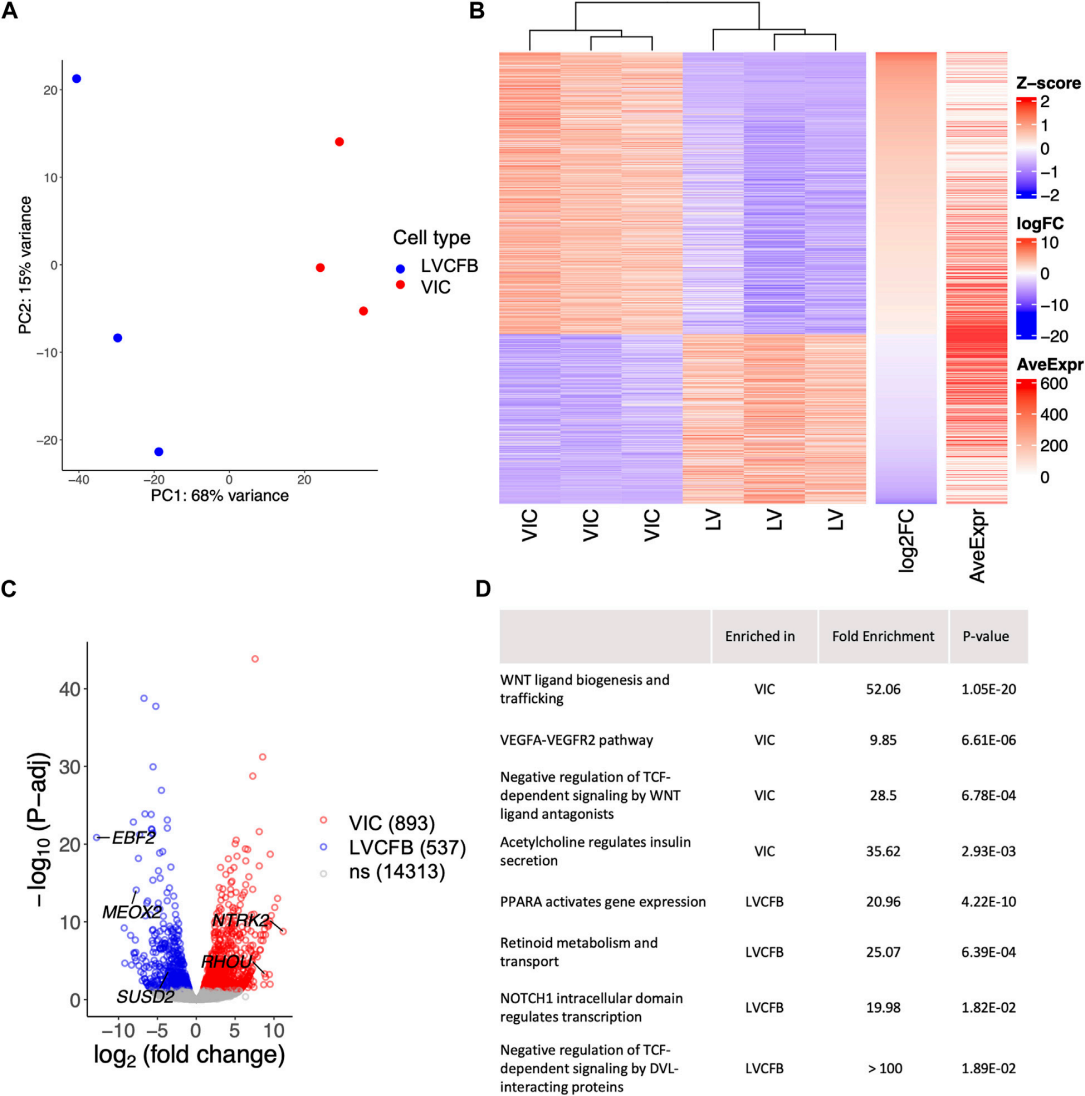
RNA-sequencing of primary CFBs. **(A)** Principal component analysis (PCA) plot of human primary LVCFB and VIC samples showing distinct clusters by cell type. **(B)** Heatmap visualizing the z-score distribution across each row (gene) in VIC and LVCFB samples with log-2-fold change and average expression displayed on right bars. **(C)** Volcano plot displaying selected differentially regulated genes including genes with the highest fold change, including *NTRK2* and *EBF2*, and *SUSD2* across log-2-fold change on the *x*-axis and -log10(P-adj) on the *y*-axis with identified potential genes that distinguish VICs and LVCFBs labelled. **(D)** Selected pathways of interest from Gene Ontology (GO) enrichment analysis with the Bonferroni corrected *p*-values of the upstream regulator genes obtained from QuatenaryProd show common developmental pathways being differentially regulated between primary CFBs. Cells in this experiment were passage 1. Full list of GO analysis results can be found in Supplementary Material S2.

To assess transcriptional differences between VICs and LVCFBs, we collected mRNA from paired donor samples and performed bulk RNA sequencing. Analysis of variance shows the two cell types clustered distinctly (Figure 1A). To identify the top differentially expressed genes driving this variance, we performed multi-factor DESeq2 to account for matched donor samples and added a threshold of p-adjusted<0.05, identifying 1,430 genes significantly differentially expressed between the VIC and LVCFB samples (Figure 1B). We then sorted the matrix by log-2-fold change and chose top genes that had high average expression and low variability in TPM values between donors to predict marker genes of each cell type (Figure 1C). Interestingly, we identified that Mesenchyme Homeobox 2 (*MEOX2*), a transcription factor that regulates transition from myofibroblasts to fibroblasts (Cunnington et al., 2014), and EBF Transcription Factor 2 (*EBF2*), a transcription factor highly expressed in the heart, were enriched in LVCFBs compared to VICs. We identified *NTRK2* and Ras Homolog Family Member U (*RHOU*) as enriched in VICs compared to LVCBs. *In vivo*, *RHOU* is expressed in the atrioventricular canal during heart development and loss of *RHOU* was shown to cause cardiac looping defects (Dickover et al., 2014).

These genes were validated using RT-qPCR (Supplementary Figure S3B) and we identified *MEOX2* and *EBF2* to be expressed at a greater level in LVCFBs than in VICs. Although the expression levels of *NTRK2* and Ras Homolog Family Member U (*RHOU*) were higher in VICs compared to LVCFBs, we did not observe a statistically significant difference to demonstrate utility as a univariate marker. However, when the ΔCt values were analyzed using a multivariate correlation analysis (Supplementary Figure S3C), we observed that paired groups of two genes (*EBF2* and *RHOU*, or *MEOX2* and *RHOU*) were statistically significant, suggesting they can be used collectively for distinguishing between VIC and LVCFB subpopulations. To validate these marker genes, we assessed their expression in publicly available single cell and bulk RNA sequencing datasets that profiled VICs and LVCFBs (Supplementary Figure S5) (Zhang et al., 2019; Tucker et al., 2020; Floy et al., 2021; Decano et al., 2022; Wang et al., 2022). We found that VICs exhibit low expression ratios of *EBF2/RHOU* and *MEOX2/RHOU* compared to LVCFB. From the single nuclei RNA sequencing results, we observed heterogeneity in the expression levels of *EBF2/RHOU* and *MEOX2/RHOU* across the different myocardial fibroblast clusters (Supplementary Figure S5F), but these ratios were consistently higher than the VIC population expression, further supporting that these genes can be used as transcriptional markers between VICs and LVCFBs in human samples.

To explore pathways that regulate the differentially expressed genes in VICs and LVCFBs, we performed QuaternaryProd analysis to predict upstream regulators of the differentially expressed genes. We identified a list of upregulated and downregulated regulator genes in VICs compared to LVCFBs (Supplementary Material S2) and performed GO enrichment analysis. We list selected biological processes in Figure 1D (full list available in Supplementary Material S2). Of note, key pathways involved in developmental regulation, including WNT, VEGF and NOTCH were identified.

### 3.3 Functional characterization of primary CFBs reveals distinct CFB signatures

#### 3.3.1 FB activation and collagen contraction assays reveal similar activation and contraction potential in LVCFBs and VICs

**FIGURE 2:**
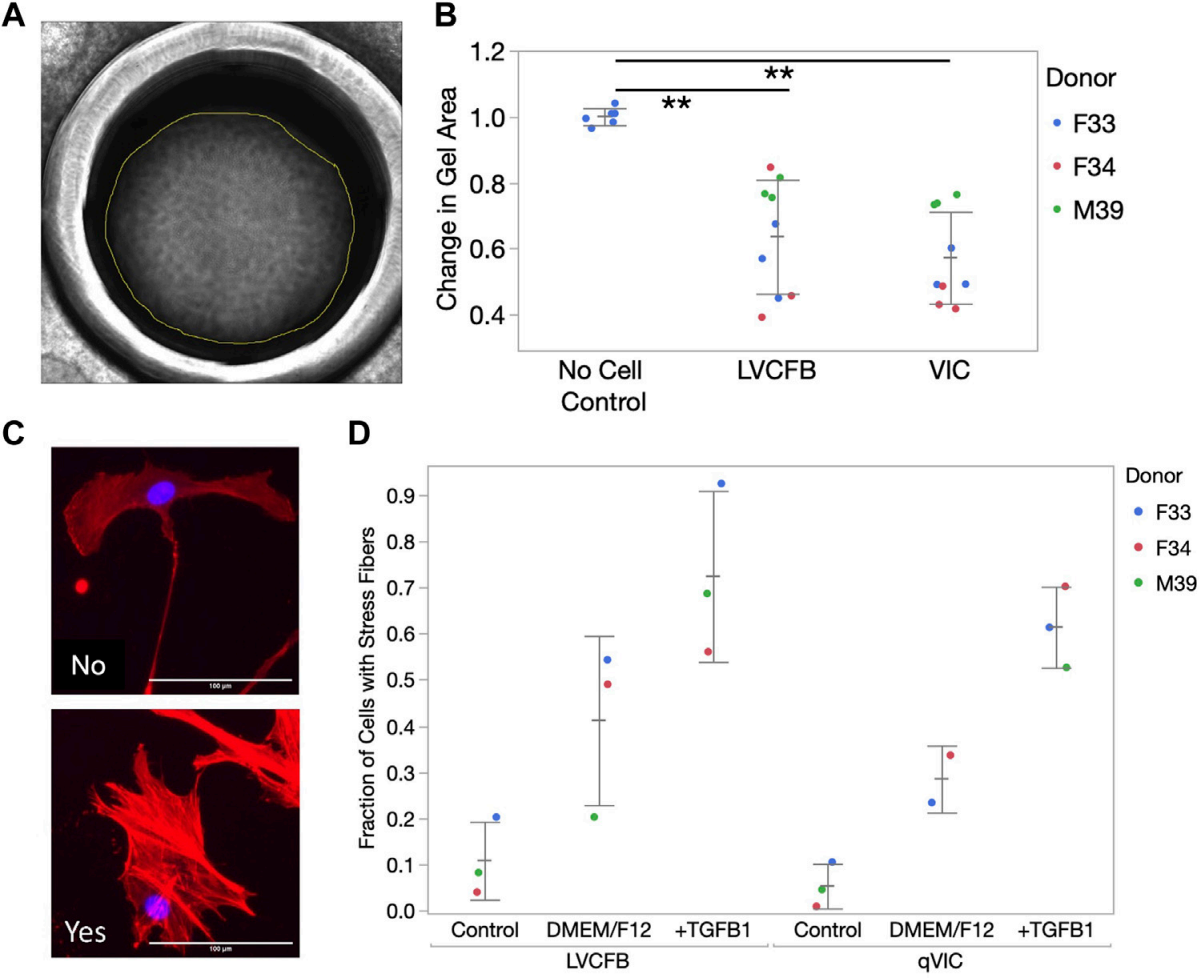
CFB contraction and stress activation. **(A)** CFB contractility was assayed through collagen gel contraction using passage 3 LVCFBs and VICs. Example brightfield image of collagen gel 24 h post-seeding. Gel area was analyzed using ImageJ. **(B)** Quantification of collagen gel contraction; gel areas with samples were normalized to an averaged area of control gels lacking cells at 24 h. Dots represent 3 wells and color represents donor. Statistics are a two-way ANOVA with Tukey’s *post hoc* test where * is *p* < 0.05 and ** is *p* < 0.01. **(C)** Primary CFB stress activation potential measured by treatment of passage 4 cells with control primary CFB maintenance medium, DMEM/F12 medium, or DMEM/F12 medium supplemented with 10 ng/mL TGFβ1. Example images of cells stained for phalloidin (red) and Hoechst (blue) that were categorized as containing stress fibers (yes) or not containing stress fibers (no). Scale bar is 100 µm. **(D)** Quantification of fraction of cells containing stress fibers. Dots represent the percentage of cells for each sample with 20–750 cells per condition and color represents the donor. Statistics are a four-way ANOVA on donor, cell source, medium, and cell source cross medium. Statistically significant increase in percentage cells with filaments in different medium (*p* < 0.05) but no difference in stress activation potential was observed between LVCFBs and VICs (*p* > 0.05).

We predicted that VICs and LVCFBs would have distinct functional properties, including contraction, activation, and mineralization potential due to their different niche environments *in vivo*. Contraction capability was assessed using a collagen gel assay. One day after seeding collagen gels in primary CFB maintenance medium, VIC and LVCFB-containing gels were smaller than no cell control gels, but there was no difference in contraction between gels seeded with the fibroblast populations (Figures 2A, B). To assess activation potential, we treated CFBs with maintenance medium, DMEM/F12, or DMEM/F12 with 10 ng/mL TGFβ1 for 2 days and then stained with phalloidin to identify formation of actin stress fibers. We acquired three images per well with four technical well replicates per given cell type from a donor. Using blinded image analysis, we classified whether each cell contained stress fibers and performed statistics on the averages for each donor. We observed increased stress fiber formation in DMEM/F12 compared to control maintenance medium and further increases upon addition of TGFβ1 in both cell types, however we did not observe a difference in activation potential between the two CFB populations (Figures 2C, D). Overall, this suggests that LVCFBs and VICs have a similar contraction potential and activation response to stress conditions induced by DMEM/F12 and TGFβ1.

#### 3.3.2 CFB mineralization reveals greater potential in LVCFBs compared to VICs under metastatic conditions

Pathologically, mineralization has been associated with valvular, coronary artery and conduction system disease, however recent studies have suggested that mineralization can also occur in the myocardium (Ahmed et al., 2020). CFBs isolated from human and mouse tissues have been shown to mineralize *in vitro* upon exposure to osteogenic medium, and *in vivo* mouse CFB calcification is thought to be regulated by the ENPP1-PPi-Pi axis (Pillai et al., 2017). Thus, we hypothesized that VICs and LVCFBs might have different mineralization potential *in vitro.*

**FIGURE 3:**
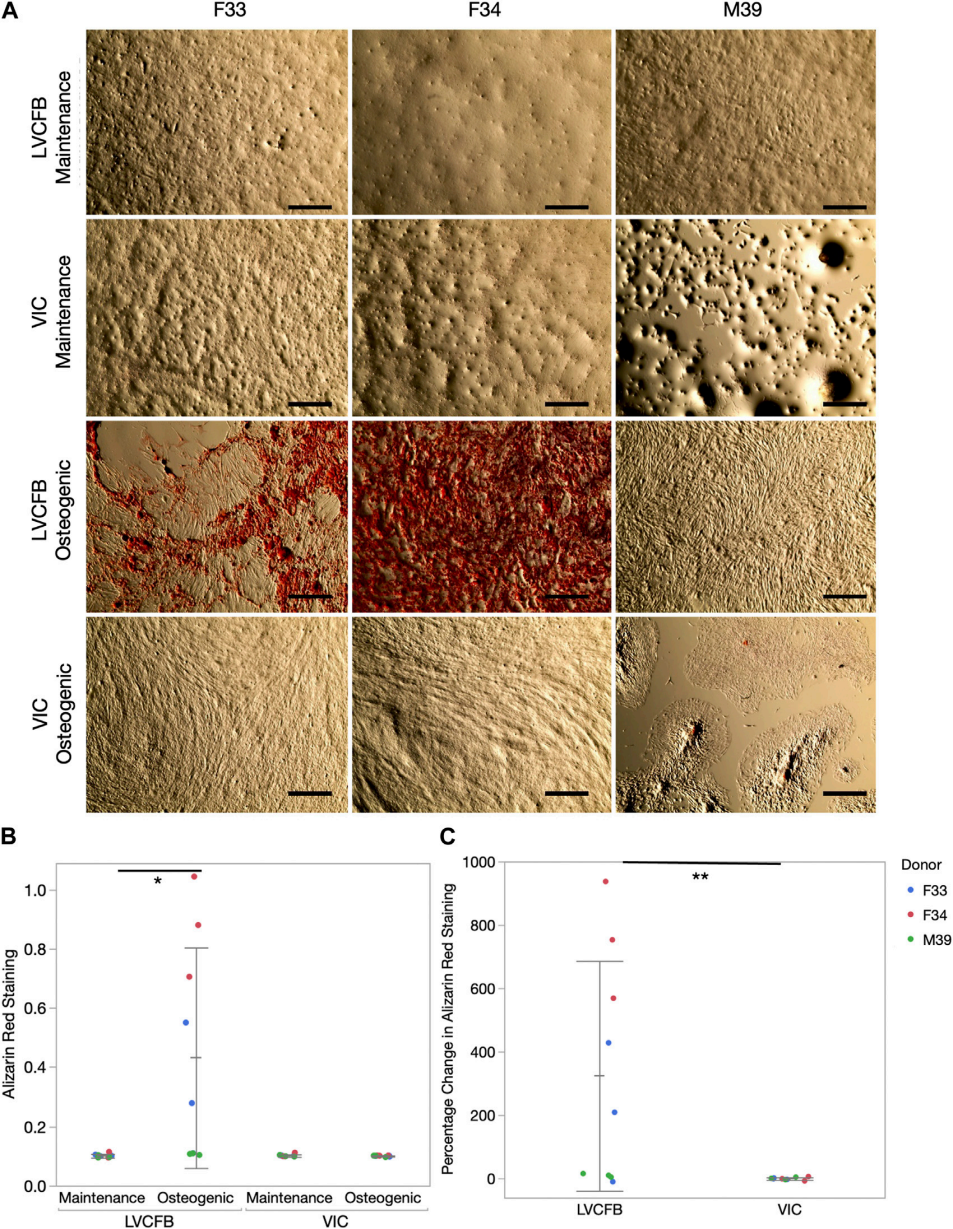
Alizarin red mineralization staining of primary CFBs. **(A)** Brightfield images of primary CFBs (passage 4) stained with Alizarin Red after 4 weeks of treatment with maintenance or osteogenic medium. Images shown are representative of three well replicates. Scale bar is 250 μm. **(B)** Quantification of Alizarin red staining by measuring absorbance at 560 nm. Dots represent three well replicates and donors are represented by color. Statistics are a two-way ANOVA on media controlling for donor where * is *p* < 0.05. **(C)** Percentage change in Alizarin Red staining comparing osteogenic to maintenance medium where dots represent three well replicates and donors are represented by color. Statistics are a two-way ANOVA on cell source controlling for donor where ** is *p* < 0.01.

**FIGURE 4:**
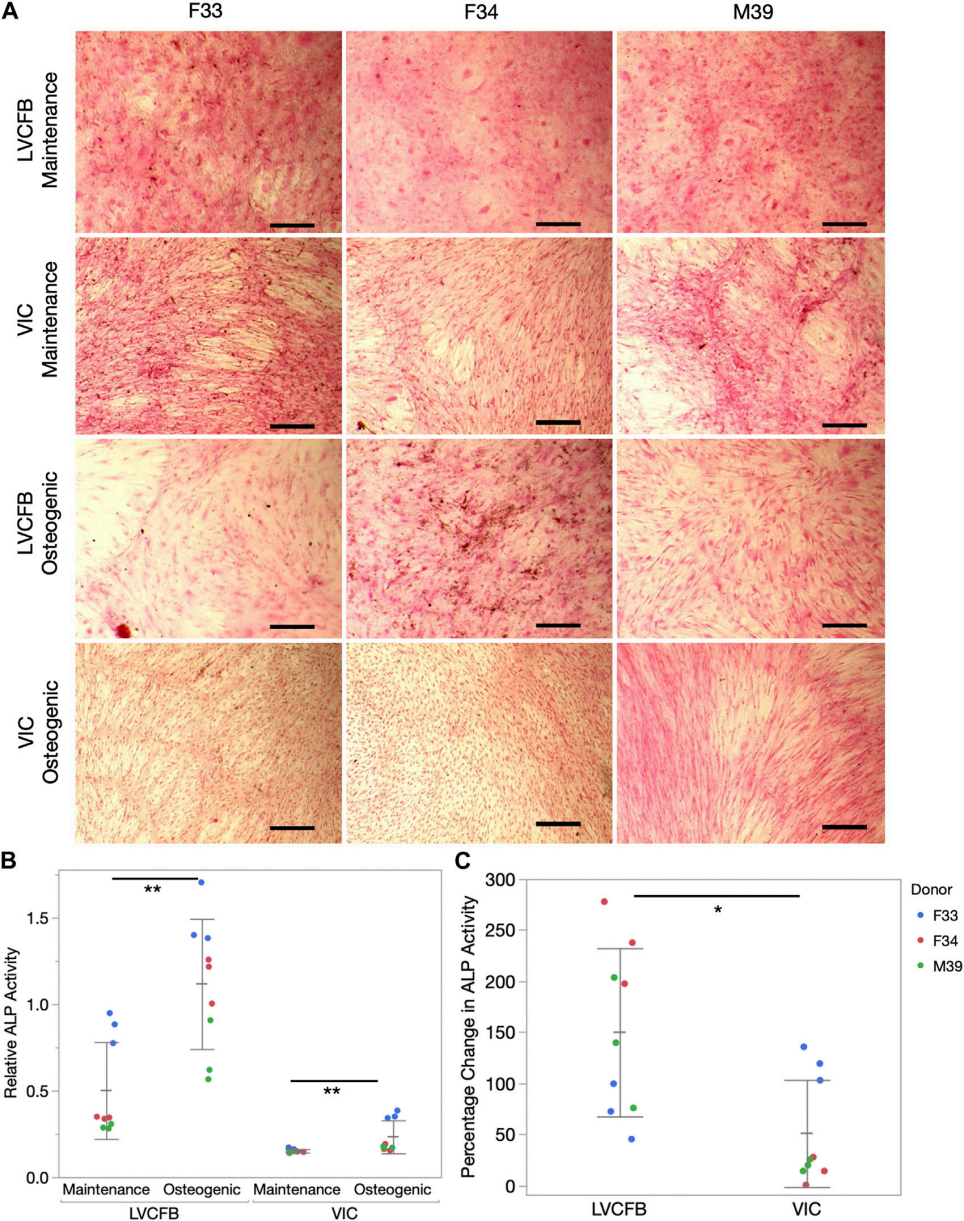
Calcification of primary CFBs. Primary CFBs (passage 4) were treated with maintenance or osteogenic medium for 4 weeks. **(A)** Von Kossa and Nuclear Fast Red Staining of primary CFBs. Calcium in mass deposits are black, calcium in dispersed deposits are gray, nuclei are red, and cytoplasm is light pink. Images are representative of three well replicates and scale bar is 100 μm. **(B)** Relative ALP activity in primary CFBs is quantified by measuring absorbance at 405 nm. Dots represent well replicates and color represents donor. Statistics are a two-way ANOVA for medium controlling for donor where * is *p* < 0.05 and ** is *p* < 0.01. **(C)** Percentage change in relative ALP activity between maintenance to osteogenic medium. Dots represent well replicates and color represents donor. Statistics are a two-way ANOVA for cell source controlling for donor where * is *p* < 0.05.

We treated LVCFBs and VICs at approximately 80%–90% confluency with primary CFB maintenance medium or osteogenic medium containing ascorbic acid, β-glycerophosphate, and dexamethasone. After 4 weeks, we stained for Alizarin red to detect mineralization, stained for von Kossa to detect calcification, and performed an ALP activity assay. We observed increased Alizarin red staining in the LVCFB treated with osteogenic medium compared to primary CFB maintenance medium (Figures 3A, B). We observed large donor-to-donor variability with the male M39 LVCFBs having minimal Alizarin Red staining, but due to sample size, we cannot conclude if this is due to donor-to-donor differences or sex-related differences. Furthermore, no difference in Alizarin red staining was observed in the VICs between the two medium conditions. Additionally, the LVCFBs exhibited a greater percentage change in Alizarin red staining upon osteogenic medium treatment compared to the VICs (Figure 3C). Similarly, we observed some von Kossa staining in LVCFB treated with osteogenic medium with minimal staining in all other conditions including VICs (Figure 4A) suggesting that the LVCFBs demonstrated higher mineralization and calcification potential compared to VICs.

To further assess if VICs could respond to the osteogenic medium, we assayed for alkaline phosphatase (ALP) activity which is an early mineralization marker. We observed increased relative ALP activity in the LVCFBs and VICs after exposure to the osteogenic factors (Figure 4B) and a greater percentage change in ALP activity in the LVCFBs compared to the VICs (Figure 4C). This suggests that LVCFBs exhibited greater mineralization potential in this experimental model compared to VICs supporting our hypothesis that CFBs from different regions of the heart have distinct functional phenotypes.

#### 3.3.3 CFB mineralization reveals greater potential in LVCFBs compared to VICs under dystrophic conditions

**FIGURE 5:**
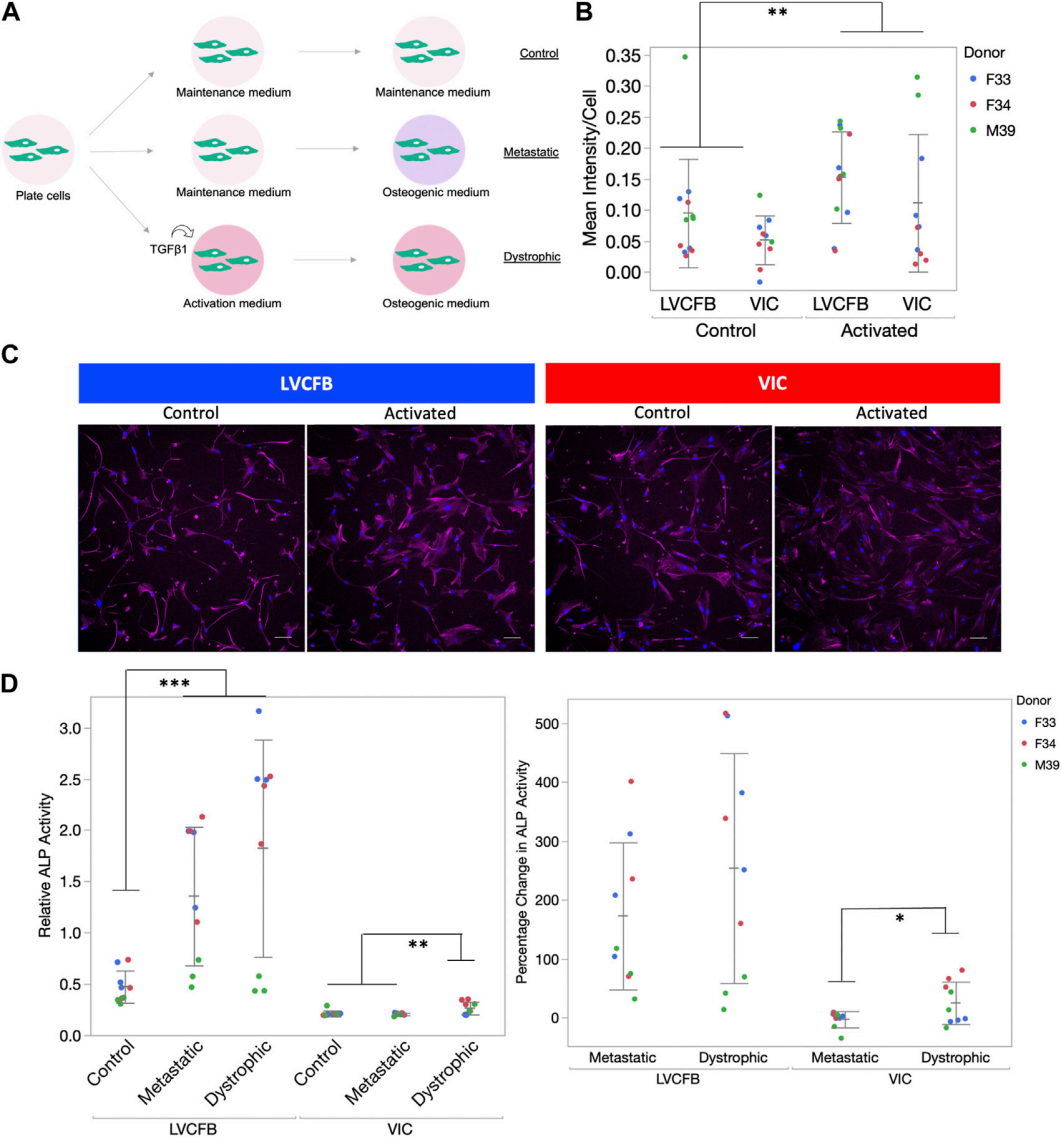
Calcification of primary CFBs post-activation. **(A)** Schematic showing experimental setup. Primary CFB samples (passages 5 and 6) were either pre-treated with activation medium or maintained in CFB maintenance medium prior to mineralization activation to mimic dystrophic and metastatic mineralization. **(B)** Quantification of αSMA immunocytochemistry (ICC) staining of cells after 3 days in activation medium. Graph is image αSMA mean intensity minus background normalized by number of nuclei. Dots represent well replicates and color represents donor. **(C)** Example ICC images used for quantification in B. Magenta is anti-αSMA antibody and blue is Hoechst stain. Scale bar is 100 μm. **(D)** Left: Relative ALP activity for primary CFBs upon activation. Dots represent well replicates and color represents donor. Right: Percentage change in relative ALP activity of metastatic and dystrophic compared to control. Dots represent well replicates and color represents donor. Statistics are a two-way ANOVA for cell source controlling for donor where * is *p* < 0.05, ** is *p* < 0.01, and ***is *p* < 0.005.

We hypothesized that the increase in LVCFB mineralization potential compared to VICs may indicate that VICs needed to undergo an intermediate stress activated state before calcification (metastatic calcification) whereas the LVCFBs may not need to be activated for higher mineralization (dystrophic calcification) (Cirka et al., 2017; Pillai et al., 2017; Li et al., 2021). To test this, we treated wells with or without activation medium containing 10 ng/mL TGFβ for 3 days before switching to osteogenic medium (Figure 5A). We confirmed cells were activated by observing higher α-SMA expression in both CFB subpopulations treated with activation media (Figures 5B, C).

Similar to data shown in Figures 4B, C, we found relative and percent ALP from control to be increased in metastatic conditions for both primary CFB populations (Figure 5D). Here, we observed a statistically significant increase in ALP activity for VICs when they were activated before treating with osteogenic factors compared to metastatic conditions. In comparison, there was no statistical difference between metastatic and dystrophic conditions for LVCFBs. This suggests that VICs are more sensitive to stress-activated dystrophic early calcification compared to LVCFBs.

#### 3.3.4 Mass spectrometry of ECM reveals the distinct matrix composition of LVCFB and VICs is consistent with the cells’ regional phenotypic function

**FIGURE 6:**
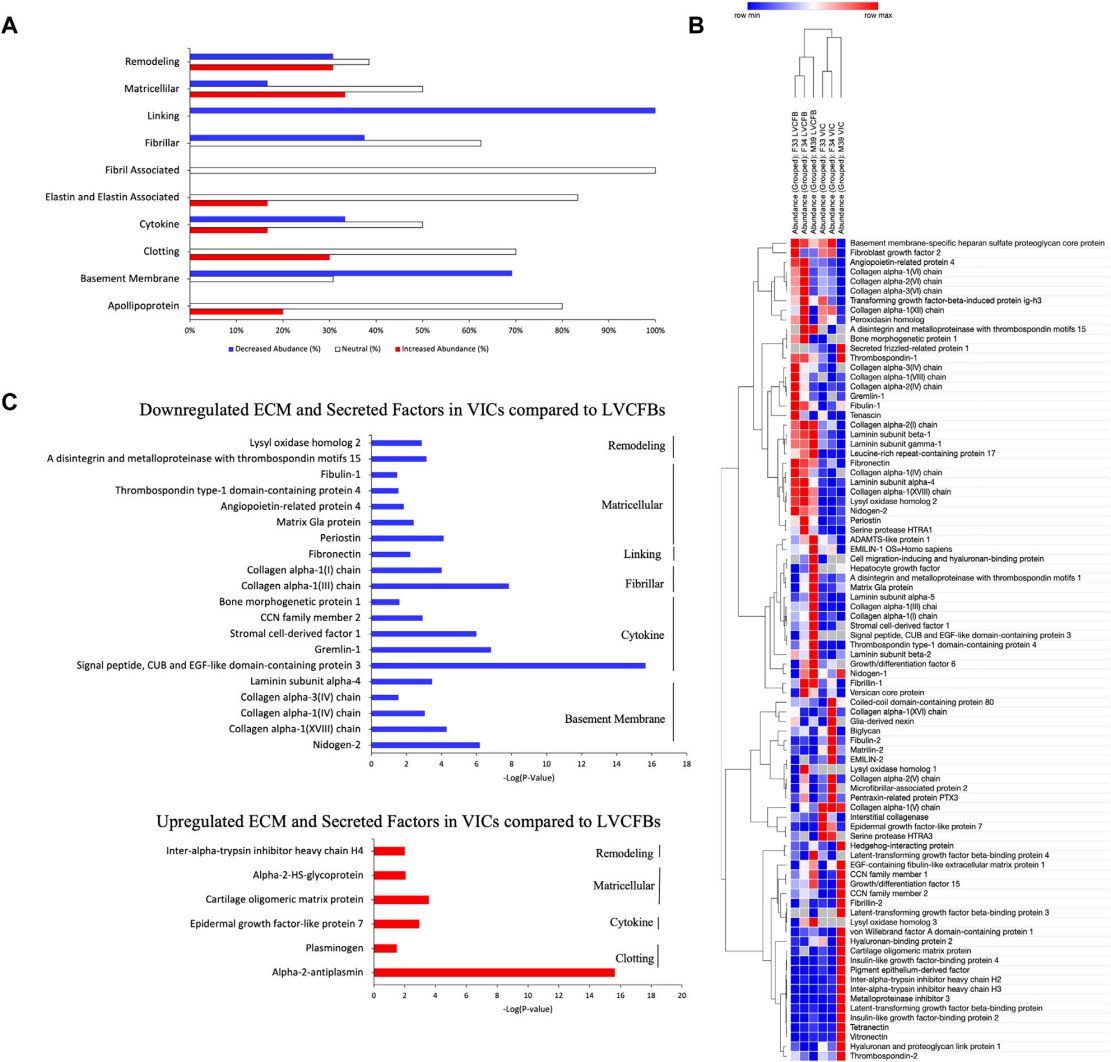
Mass Spectrometry for ECM and Secreted Factors. **(A)** The relative abundance ratio of VIC/LVCFB ECM and secreted factors as detected by mass spectrometry was used to determine the percentage of proteins detected with decreased abundance (VIC/LVCFB Abundance ratio <0.5), increased abundance (>2.0) or those that remained unchanged (0.5–2.0). Cells used in this experiment were of passages 4 and 5. **(B)** Hierarchical Clustering using the one minus Pearson’s correlation of *n* = 3 replicates from three different donors determines that the ECM and secreted factors of VICs and LVCFB cluster distinctly. **(C)** ECM and secreted factors of interest that were determined to be statistically significantly (*p* < 0.05) up or downregulated in VICs in comparison to LVCFB (*n* = 9 grouped across the three different donors).

Since LVCFB and VIC populations support different regions of the heart that have different structure and function, we hypothesized that these cell types would secrete different ECM profiles in concordance with their roles. To test this on a protein level, we cultured LVCFBs and VICs at a high density for 16 days. On day 16, we decellularized the matrices, harvested the ECM, and performed mass spectrometry to identify and analyze relative and secreted factors. Detected proteins were sorted into ten categories; remodeling, matricellular, linking, fibrillar, fibril associated, elastin and elastin associated, cytokines, clotting, basement membrane and apolipoproteins (Supplementary Material S3). The relative abundance ratio was used to determine whether each protein was more abundant (ratio > 2.0), of similar abundance (ratio from 0.5 to 2.0), or less abundant (ratio < 0.5) in VICs compared to LVCFBs (Figure 6A). Broadly, VICs had a lower deposition of linking, basement membrane proteins, and fibrillar collagens than LVCFBs. There was a slight increase in the abundance of proteins in the elastin and elastin-associated, clotting, and apolipoprotein categories in the VICs compared to the LVCFBs. Remodeling, matricellular and cytokine subclasses had various proteins that were differentially produced between both populations. Hierarchical clustering of the relative abundance of each protein for each donor determined that VIC and LVCFB matrices clustered distinctly from each other irrespective of donor (Figure 6B). Notably, the male VIC matrices and LVCFB matrices clustered distinctly from the female lines but additional donors would be needed to determine if these differences are due to sex-differences or donor related differences.

Of the 107 proteins detected, 32 had a VIC/LVCFB abundance ratio that was statistically decreased in abundance (*p* < 0.05) and only 6 were increased in abundance in the VICs, including alpha-2-antiplasmin, plasminogen, epidermal growth factor-like protein 7, cartilage oligomeric matrix protein, alpha-2-HS-glycoprotein, and inter-alpha-trypsin inhibitor heavy chain H4 (Figure 6C). This potentially indicates that fibroblasts that occupy the heart chamber play a larger role in ECM production and remodeling then valve fibroblasts in adulthood. The fibrillar collagens I and III, which are prevalent in the heart and play a role in maintaining the mechanical integrity of the ventricle were present in larger abundances in the LVCFB samples than the VIC samples (Brower et al., 2006). Additionally, the key remodeling protein, lyse oxidase homolog 2, which is important in fibrillar collagen fibril formation was also significantly upregulated in LVCFB matrices compared to VIC matrices (Rodríguez and Martínez-González, 2019). Other notable differences include the increased presence of fibronectin and periostin ECM proteins in the LVCFBs compared to VICs; both proteins play important roles in heart development and injury response (George et al., 1993; Konstandin et al., 2013; Landry et al., 2017).

## 4 Discussion

CFBs play a crucial support role in the heart by producing and organizing extracellular matrix, secreting paracrine factors, interacting with other cell types, and responding to stress stimuli. Developmental lineages and differences in the local environmental niche are thought to lead to CFB specification resulting in molecular and functional phenotypical differences in different regions of the heart. Tissue level specification may be a driver in regional-specific patient pathology and since some CFBs in the valve and most CFBs in the myocardium arise from epicardial lineages, learning the differences between regional tissues and developing protocols to specify cell types can shed light onto disease mechanism and help develop better therapeutics (Sun et al., 2013; Moore-Morris et al., 2014; Liu et al., 2018).

In this study, we compared molecular and functional attributes between three paired samples of human VICs and LVCFBs. We identified that the VICs exhibited a larger cell area, perimeter, and circularity compared to the LVCFBs. We found that both populations stained positive for common cardiac fibroblasts markers and displayed similar stress fiber activation potential and collagen gel contraction, indicating that LVCFBs and VICs possess some similar attributes.

We identified that the LVCFBs expressed SUSD2, a novel cultured CFB marker (Schmuck et al., 2021), while the VICs did not. Furthermore, from our bulk RNA-sequencing results, we identified a panel of genes, *RHOU*, *MEOX2*, and *EBF2* to distinguish between VICs and LVCFBs at a transcript-level. *MEOX2* is a transcription factor that decreases in expression during conversion of fibroblasts to myofibroblasts. Its lower expression in VICs compared to LVCFBs could indicate a difference in the susceptibility of VICs to myofibroblast activation. Not much is known about the role of *EBF2* in cardiac tissue development or homeostasis. *In vivo*, *RHOU* is expressed in the atrioventricular canal during heart development and loss of *RHOU* was shown to cause cardiac looping defects (Dickover et al., 2014). Furthermore, *RHOU* expression is regulated by Wnt1 (Tao et al., 2001) and Wnt2 signaling (Dickover et al., 2014) both of which were enriched upstream regulators in the VICs compared to LVCFBs. To further test these markers, we compared expression of these genes in publicly available bulk and single cell RNA sequencing datasets (Zhang et al., 2019; Tucker et al., 2020; Floy et al., 2021; Decano et al., 2022; Wang et al., 2022). We show our marker genes show similar trends in these datasets, as our calculated *EBF2/RHOU* and *MEOX2/RHOU* ratios are consistently low in the VIC datasets and high in the LVCFB datasets. These fold-level statistically significant differences in transcriptional expression show robust validation of these genes as a method to identify regional specification of these subpopulations.

Since assessment of these markers at the protein level would be useful in discriminating fibroblast populations, we attempted to validate expression of EBF2, RHOU2, and MEOX2 in both primary human LVCFBs and VICs, and in primary porcine tissue samples of the left ventricle and aortic valves *via* immunostaining and Western blotting. However, the antibodies we were able to obtain provided no signal in samples or positive controls, or exhibited high non-specific background (e.g., signal in negative controls or non-nuclear signal) It is possible that since these genes encode transcription factors, their low protein levels may not make them suitable targets for protein-level discrimination. Based on these results, we cannot currently validate the ability of proteins encoded by *EBF2*, *RHOU2*, and *MEOX2* to discriminate between LVCFs and VICs, but identification of proteins that can discriminate between fibroblast populations would facilitate analytic efforts to assess fibroblast identity and heterogeneity.

Since calcification is a significant problem in valve disease pathology, we were surprised to find LVCFBs exhibited higher mineralization and calcification potential compared to VICs. However, in a dystrophic condition, activated VICs displayed increased ALP activity compared to a metastatic condition, whereas LVCFBs displayed similar ALP activity regardless of an intermediate activated stressed state, indicating these fibroblast subpopulations may differ in mechanism of early calcification. Understanding the differences in calcification mechanism may provide an avenue for development of regional pathology specific anti-calcification drugs. To note here, we consider the VICs used in this study to be mostly quiescent as observed through morphology and low fraction of cells exhibiting stress fibers. Additionally, we used a medium formulation that has been shown to keep VICs cells in a quiescent phenotype (Porras et al., 2017). Cells in the calcification post-activation experiment expressed aSMA in the control condition which could indicate a stress response to a higher passage number. However, since there was a significant difference in aSMA expression between control and activated conditions, we can conclude the VICs were responsive to activation stimuli.

We compared ECM protein synthesis between VICs and LVCFBs using mass spectrometry and found that VICs secreted less ECM proteins compared to ventricular CFBs. In fact, only 6 proteins were significantly increased in abundance in the VIC samples and they belonged to clotting, cytokine, matricellular and remodeling subclasses. While our mass spectrometry results included proteins reported to be found in valves such as collagen type I and III or periostin (Lockhart et al., 2011; Wiltz et al., 2013), we found that their abundance in LVCFB matrices was much greater than in VIC matrices. These give an insight into functional necessity as collagen fibers mainly function for tensile strength and flexibility, and since the left ventricle experiences the highest systolic pressure, this could explain the increased secretion by ventricular CFBs compared to valve CFBs (Wiltz et al., 2013). Similarly, periostin has been shown to promote cardiomyocyte proliferation after injury and this function is useful in the myocardium (Polizzotti et al., 2012). Cartilage oligomeric protein (COMP) and alpha 2-Heremans-Schmid glycoprotein (AHSG) were among the 6 VIC upregulated proteins. COMP has been reported to be expressed higher in calcific valve samples near calcific nodules in calcific aortic valve disease patients (Xu et al., 2021) and AHSG was shown to be an inhibitor of ectopic calcification (Schäfer et al., 2003). Furthermore, we also found some previously identified valve ECM proteins, including hyaluron-1 and fibrillin-2, to have a VIC/LVCFBs abundance ratio greater than 1, but these showed variability in magnitude between donor samples (*p*-values 0.25 and 0.77 respectively) (Lockhart et al., 2011; Wiltz et al., 2013).

Cellular sex is important to consider in basic cardiac research (Ventura-Clapier et al., 2017) and may play a key role in CFB specification. For example, porcine VICs have sex-dependent angiogenic secretion profiles (Nelson et al., 2021) and mice have sex-dependent fibrosis pathologies (Achkar et al., 2020). Furthermore, clinical differences in valve calcification between sexes have been reported and has been a recent interest in the field (Myasoedova et al., 2020; Aguado et al., 2022). We observed our 2 female donors clustered together in the mass spectrometry results regardless of cell type, but due to sample size limitations, we are unable to ascertain how sex differences affect ECM secretion in this study. Additional limitations of our study regarding donor samples include similar age and a history of hypertension. All three donors in this study were of similar young age (33, 34, and 39 years old) and while these patients were considered healthy, they had a previous history of hypertension. We need to consider that some findings of this study may differ with older donors or patients without hypertensive history. To test if these limitations affected the expression of identified marker genes, we first compared the *EBF2/RHOU* and *MEOX2/RHOU* ratios between cultured human fetal and adult ventricular CFBs from a publicly available RNA sequencing dataset (Floy et al., 2021) to assess the effect of developmental stage. Here, we noted no statistical differences between the fetal and adult groups suggesting these gene expression ratios are not age dependent (data not shown). To test if these gene expression ratios are dependent on a diseased state, we compared these ratios from a single cell study using ventricular and intraventricular septum cells from mice undergoing sham and MI-induced surgery procedures (Farbehi et al., 2019). Here, we obtain four fibroblast clusters, and we found the *MEOX2/RHOU* and *EBF2/RHOU* TPM averages in a sham, 3 days post-surgery and 7 days post-surgery conditions were 4.2, 5, and 2.2 respectively for *MEOX2/RHOU* and 1.5, 2.2, and 0.8 respectively for *EBF2/RHOU* (data not shown). The dropouts in the single cell RNAseq datasets suggest low expression or insufficient sequencing depth, but this variability in expression ratios between days 3 and 7 post-surgery suggest these markers may be dependent on recovery processes following MI. However, this needs to be further studied.

Human pluripotent stem cells (hPSCs) provide an alternative to primary cells for *in vitro* modeling and *in vivo* cell therapy applications. Due to the small amount of tissue present in the valves, isolating human VICs can be difficult as living heart tissue is hard to obtain in adults and nearly impossible to obtain in early stages of development (Scott et al., 2021). Human VICs and LVCFBs have limited expansion capability and maintaining VICs in a quiescent state has previously been a major hurdle for the field (Latif et al., 2015; Porras et al., 2017). hPSC sources offer a greater potential for manipulation, improved consistency between experiments, recapitulation of early development and early onset of disease states. However, little is known about regional specificity of fibroblasts differentiated from hPSCs (Floy et al., 2021). Findings from this study could inform methods to generate human stem cell-derived VICs or LVCFBs.

We have previously reported a protocol to generate hPSC-derived epicardial cells and their subsequent differentiation to hPSC-derived cardiac fibroblasts (EpiC-CFBs) (Floy et al., 2021). In addition, we have characterized the differences between hPSC-derived epicardial and second heart field-derived fibroblasts (SHF-CFBs) (Floy et al., 2021). Fibroblasts generated from both protocols expressed characteristic fibroblast markers and had unique gene expression signatures, assessed by bulk RNAseq (dataset available at GSE168380) (Floy et al., 2021). When we compared TPM values of the differentially expressed genes between VICs and LVCFBs in the hPSC-CFBs against the primary ventricular CFBs collected in this dataset, we find that both hPSC-derived cell populations expressed significantly lower TPM values compared to adult ventricular CFBs for the two transcriptional LVCFB markers, *MEOX2* and *EBF2* and higher expression of *RHOU* on a transcript-level compared to adult ventricular CFBs, more consistent with primary VICs than LVCFBs. This transcript level difference could suggest that current hPSC-CFBs protocols generate cells that may be VIC-like. To test proteomics, we combined mass spectrometry data from the previous study Floy et al., 2021) and this one and compared the ratio of protein expression between primary and hPSC-derived CFBs. A limitation of this analysis is that culture conditions and experimental procedure between these two studies were different and this could impact this comparative analysis. We found proteomics did not reveal a bias of hPSC-derived CFB identity toward either primary cell type (Supplementary Material S4). This could indicate that both current protocols generate hPSC-derived CFBs that may lean toward VIC-like on a transcriptional basis, but further manipulation is required to make these cell region-specific.

Identifying fate specifying pathways is a critical step in generating region-specific cells. Here, identified upstream regulators of the list of differentially expressed genes using QuartneryProd analysis with the GO database to identify differential pathways between VICs and LVCFBs. We identified various cardiac developmental pathways, including VEGF and WNT, which were enriched among the VIC upstream regulators, and the NOTCH pathway was enriched among the LVCFB upstream regulators. Multiple canonical and non-canonical Wnt pathways are involved in endothelial-to-mesenchymal (EndoMT) transition, proliferation of the endocardial cushion stage in development and in osteoblast differentiation (Alfieri et al., 2010). In adults, Wnt pathways are involved in regulating fibrosis and are quiescent until they are reactivated in various cardiac diseased conditions (Dawson et al., 2013). In the valves, Wnt upregulation has been shown to worsen pathogenesis of aortic valve stenosis (Khan et al., 2020). VEGF signaling is involved in EndoMT in the outflow tract regions and through VEGFR2 is involved in the maturation of atrioventricular cushions into leaflets (Stankunas et al., 2010). In adults, VEGF was shown to be involved in vascular homeostasis and to promote angiogenesis in myocardial infarcted rats (Lee et al., 2007; Lv et al., 2018), but little is understood about its role in adult valves apart from its significance in development. During valve formation, Notch signaling activates the epithelial-to-mesenchymal transition and mutations results in defective valves (MacGrogan et al., 2011). Furthermore, suppression of Notch signaling has been shown to increase calcific nodules in VIC cultures suggesting a homeostatic role in adulthood (Nisham and Srivastava, 2009). The Notch signaling pathway is also involved in fibrosis, cardiac hypertrophy, and regenerative process after injury (Nemir et al., 2014; Nistri et al., 2017). These critical pathways have been leveraged to generate valve endothelial cells and subsequently VICs from hPSCs in a previous study by treating cardiac progenitor cells with VEGF, TGFβ1, BMP4, and bFGF (Cheng et al., 2021) and may be promising to explore in generating a differentiation protocol from hPSC-derived epicardial cells to VICs.

In summary, CFB subtype specification highlights the diversity and complexity of cardiac cell subpopulations. By directly comparing CFBs isolated from the left ventricle and the aortic heart valve, we have identified key molecular and functional phenotypes which can be used to further understand regional disease pathology.

## Data Availability

The RNA sequencing data presented in the study are deposited in the Gene Expression Omnibus (GEO) repository, accession number GSE218251.

## Supplementary material

Supplementary Table S1 Processed RNA sequencing results.

Supplementary Table S2 Mass spectrometry results of primary CFBs.

Supplementary Table S3 Mass spectrometry results comparing hPSC-derived and primary CFBs.

Supplementary Presentation 1 Supplementary Figures and Tables.

## Abbreviations

ALP, alkaline phosphatase; CFB, cardiac fibroblast; ECM, extracellular matrix; hPSC, human pluripotent stem cell; LVCFB, left ventricular cardiac fibroblast; VIC, valve interstitial cell.
