# Supplementary material for: Identifying molecular and functional similarities and differences between human primary cardiac valve interstitial cells and ventricular fibroblasts
Source: Front Bioeng Biotechnol. 2023 Mar 27;11:1102487. doi: 10.3389/fbioe.2023.1102487 (PMC10083504; doi:10.3389/fbioe.2023.1102487)
Supplement: Supplementary file 3 [file Presentation1.pdf]

Supplementary Material

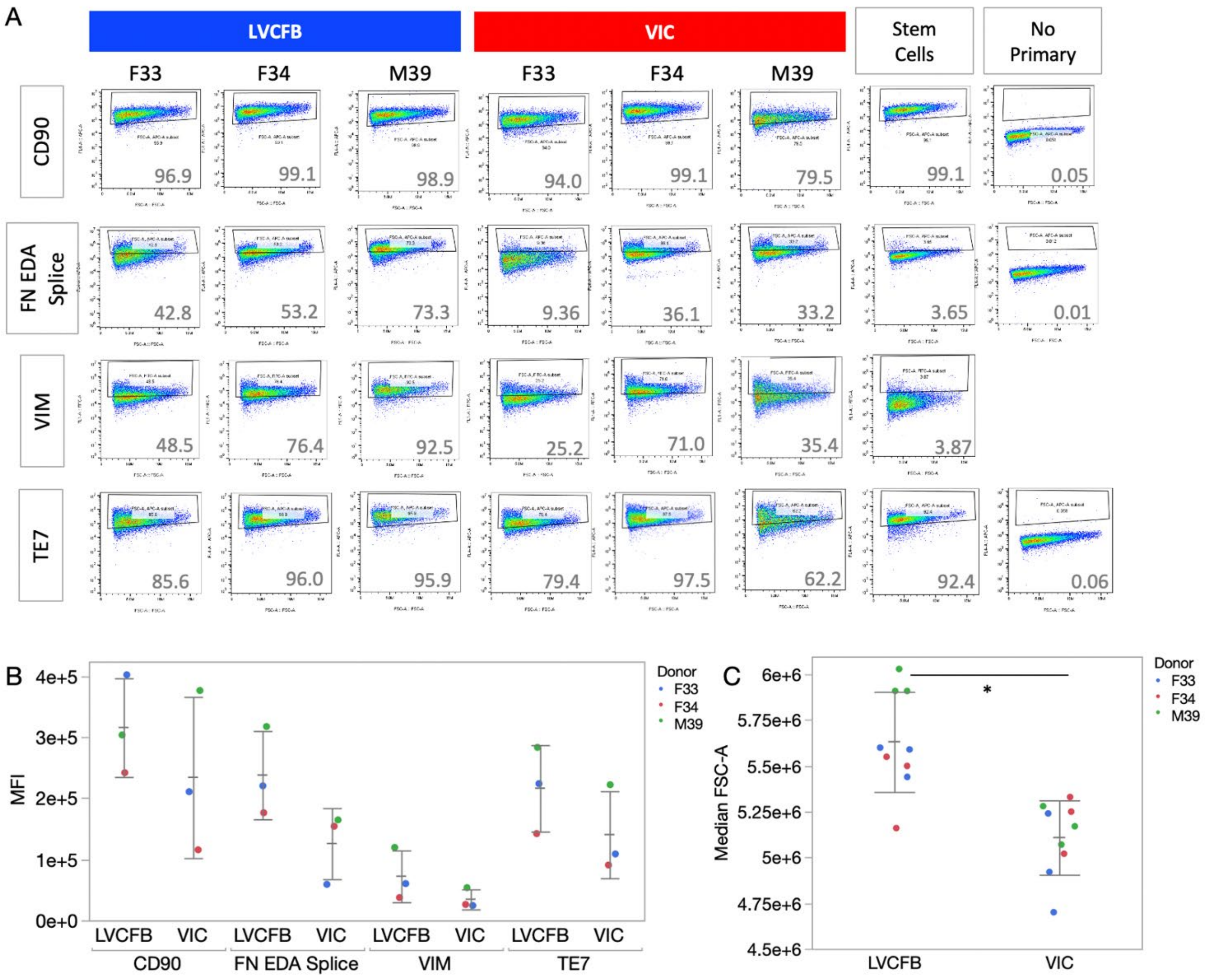

Figure S1: Flow cytometry gating plots.

(A) Flow cytometry staining of primary CFBs (passage 1) for CD90, FN EDA splice variant, VIM, and TE7. Gating based on undifferentiated hPSCs (H9 stem cells) and no primary antibody controls. Values in grey represent percent of cells in gated samples; x-axis is FSC-A and y-axis is FITC-A or APC-A accordingly. **(B)** Mean fluorescent intensities of common fibroblast marker proteins from panel A in primary CFB subpopulations. No statistical difference in values between subpopulations based on

paired Student's t-test. **(C)** Median FSC-A of primary CFBs stained with phalloidin for cell size comparison. Dots represent well replicates and color represents donor. Statistics are a two-way ANOVA where ** is P<0.01.

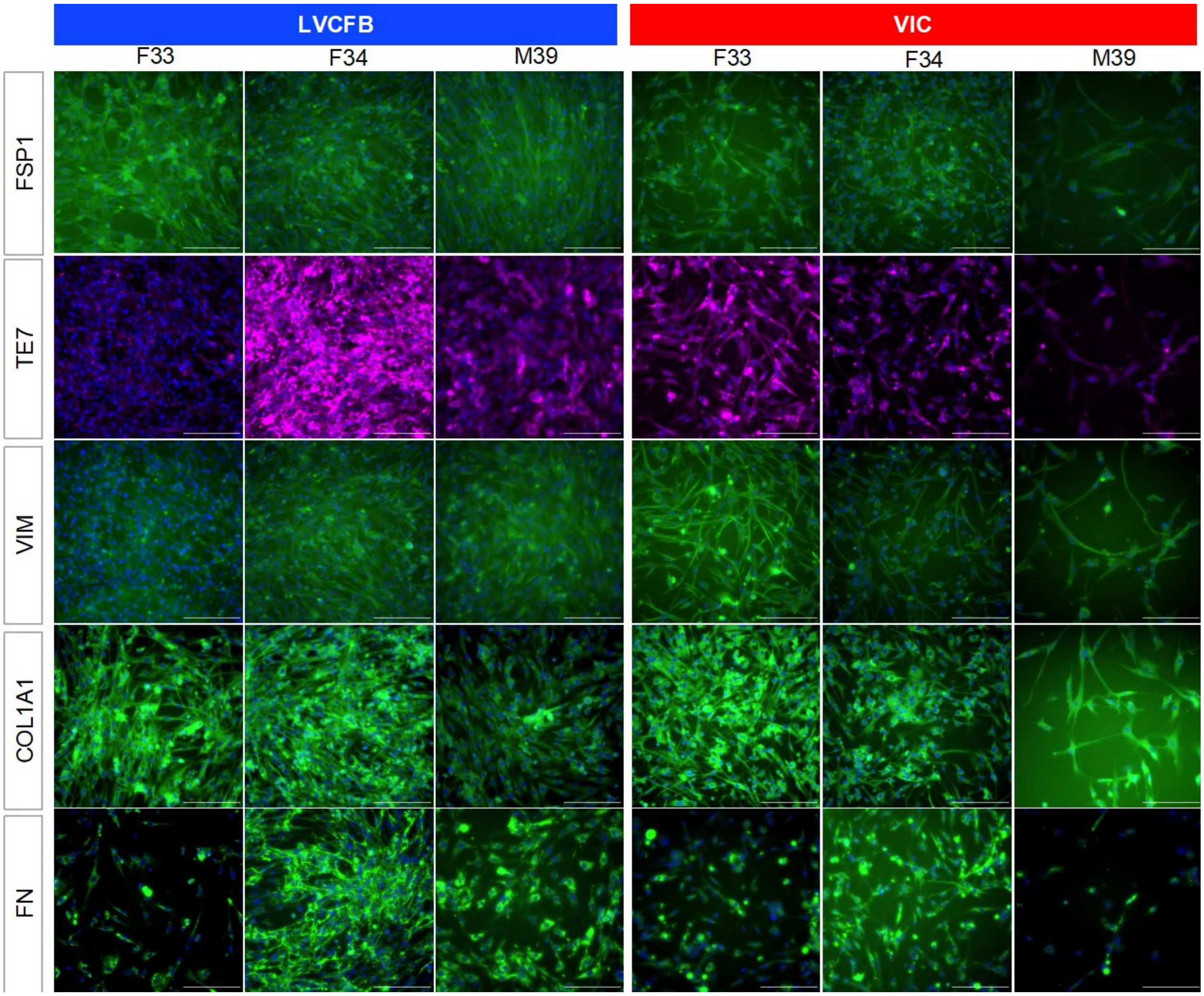

Figure S2: Immunofluorescence staining for CFB markers in primary CFBs.

Immunocytochemistry staining of primary CFBs (passage 1) for fibroblast proteins (cyan and green) and co-stained with Hoechst nuclear stain (blue). Cells were cultured in maintenance medium, fixed in 4% PFA prior to staining and imaged using an epifluorescence microscope. Scale bars are 200 µm.

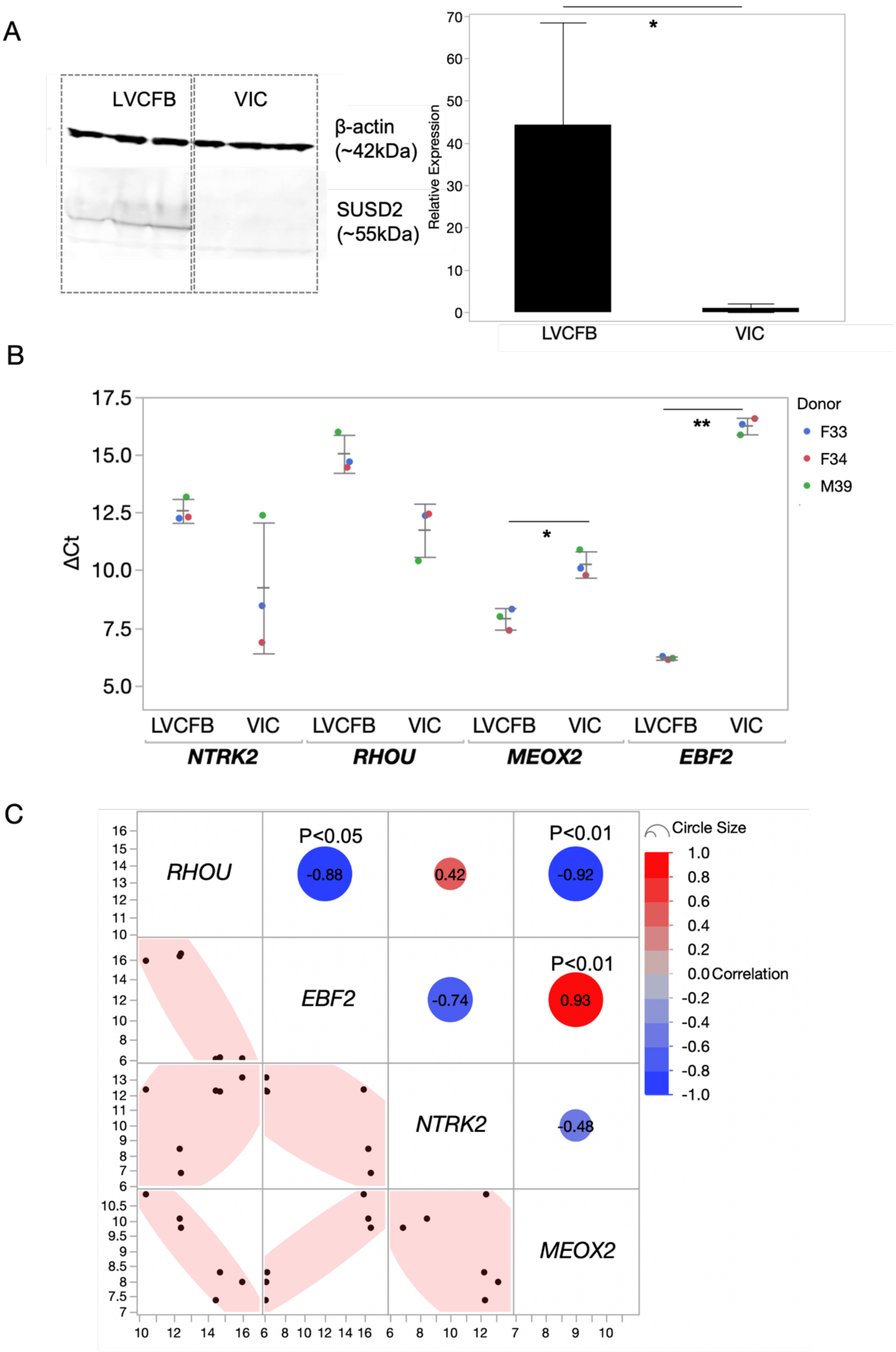
A
LVCFB
VIC
β-actin (~42kDa)
SUSD2 (~55kDa)
Relative Expression
70
60
50
40
30
20
10
0
*
LVCFB
VIC
B
ΔCt
17.5
15.0
12.5
10.0
7.5
5.0
**
*
Donor
F33
F34
M39
LVCFB
VIC
NTRK2
RHOU
MEOX2
EBF2
C
RHOU
EBF2
NTRK2
MEOX2
P<0.05
-0.88
0.42
P<0.01
-0.92
-0.74
P<0.01
0.93
-0.48
Circle Size
Correlation
1.0
0.8
0.6
0.4
0.2
0.0
-0.2
-0.4
-0.6
-0.8
-1.0

Figure S3: Western blot and RT-qPCR of identified genes

(A) Left: Western blot of SUSD2 aVnd β-actin expression. N=3 biologic replicates (one sample per donor). Right: Bands were quantified to calculate relative expression of SUSD2 normalized to β-actin. Bands normalized to β-actin expression. Statistics are a paired student's t-test where * is $P<0.05$ **(B)** Expression of genes identified from DESeq assessed by RT-qPCR. Each dot represents an average of 3 well replicates with the exception of F34 LVCFB (2 replicates). Y-axis is ΔCt calculated by subtracting Ct value of gene of interest by the Ct value of the housekeeper gene (*RPL13A*) in each sample. Statistics are a paired student's T-test where * is $P<0.05$, and ** is $P<0.001$. **(C)** Multivariate correlation matrix containing Ct values across x- and y- axes. Circle size and color represent correlation size and direction respectively. Shaded ellipses display 90% confidence interval. Pairwise correlations p-values are displayed above circles; non-significant correlations are not displayed.

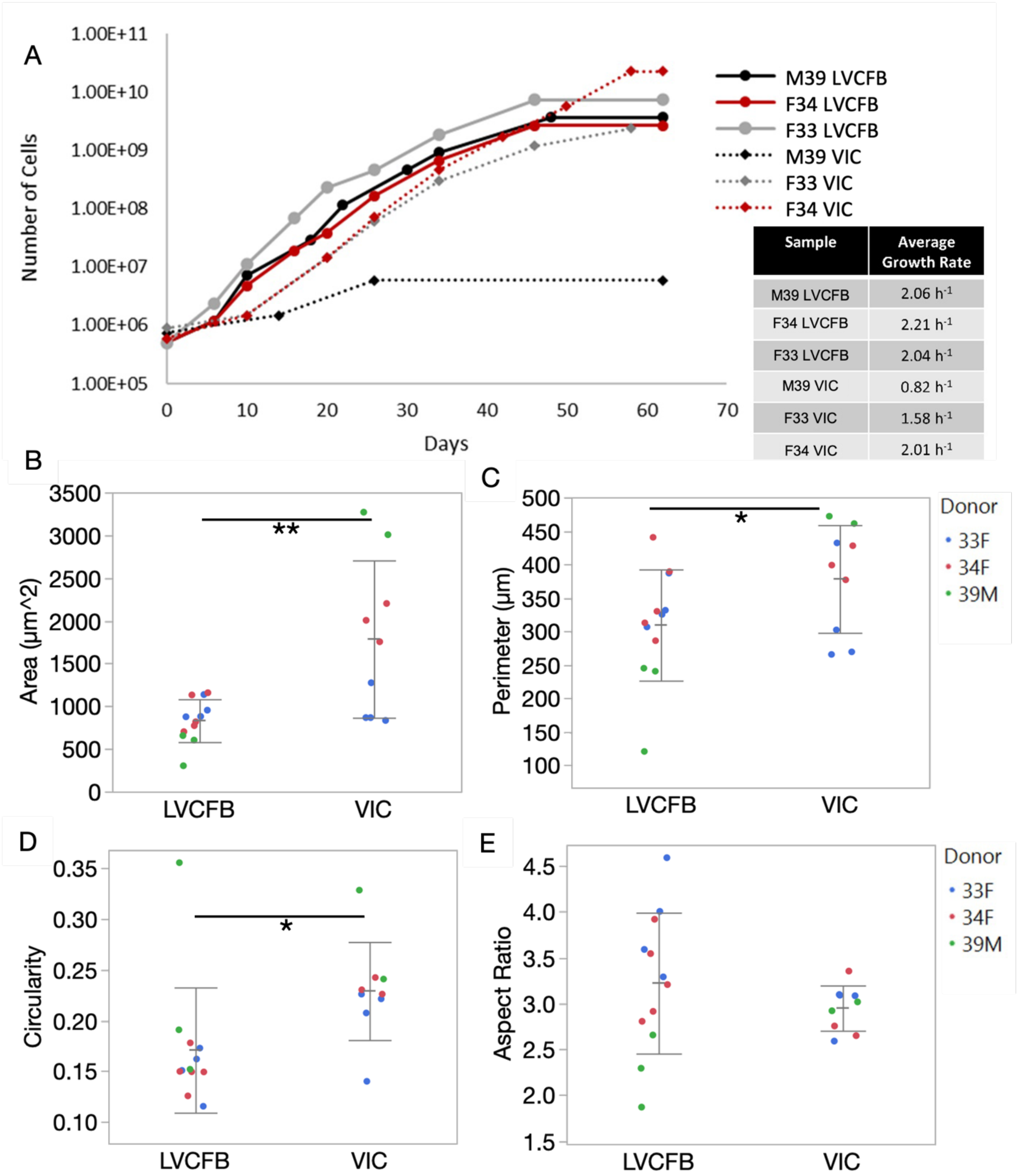

Figure S4: Primary CFB cell attributes analysis.

(A) Growth rate of primary CFBs. Cells were seeded at 6800 cells/cm^2 and passaged at 80-90% confluency. At every passage, the numbers of cells in three wells were counted using a hemocytometer and the average cell density was calculated. Passages are represented by dots. We did not maintain

well replicates since donors were our technical replicates for statistical analysis. No statistically significant difference (P>0.05) by a paired Student's t-test in the growth rate (slope of total number of cells versus time). Additionally, a two-way ANOVA for cell type and donor showed that there was no statistical difference amongst donors (P>0.5). **(B-E)** Image-based analysis of area, perimeter, circularity, and aspect ratio cell shape parameters. Primary CFBs were stained with phalloidin and analyzed using thresholding in ImageJ. Cells from 2-5 wells from each do nor are shown with at least 80 cells per well. Dots represent well replicates and color corresponds to donor. Statistics are a paired Student's t-test where * is P<0.05 and ** is P<0.01.

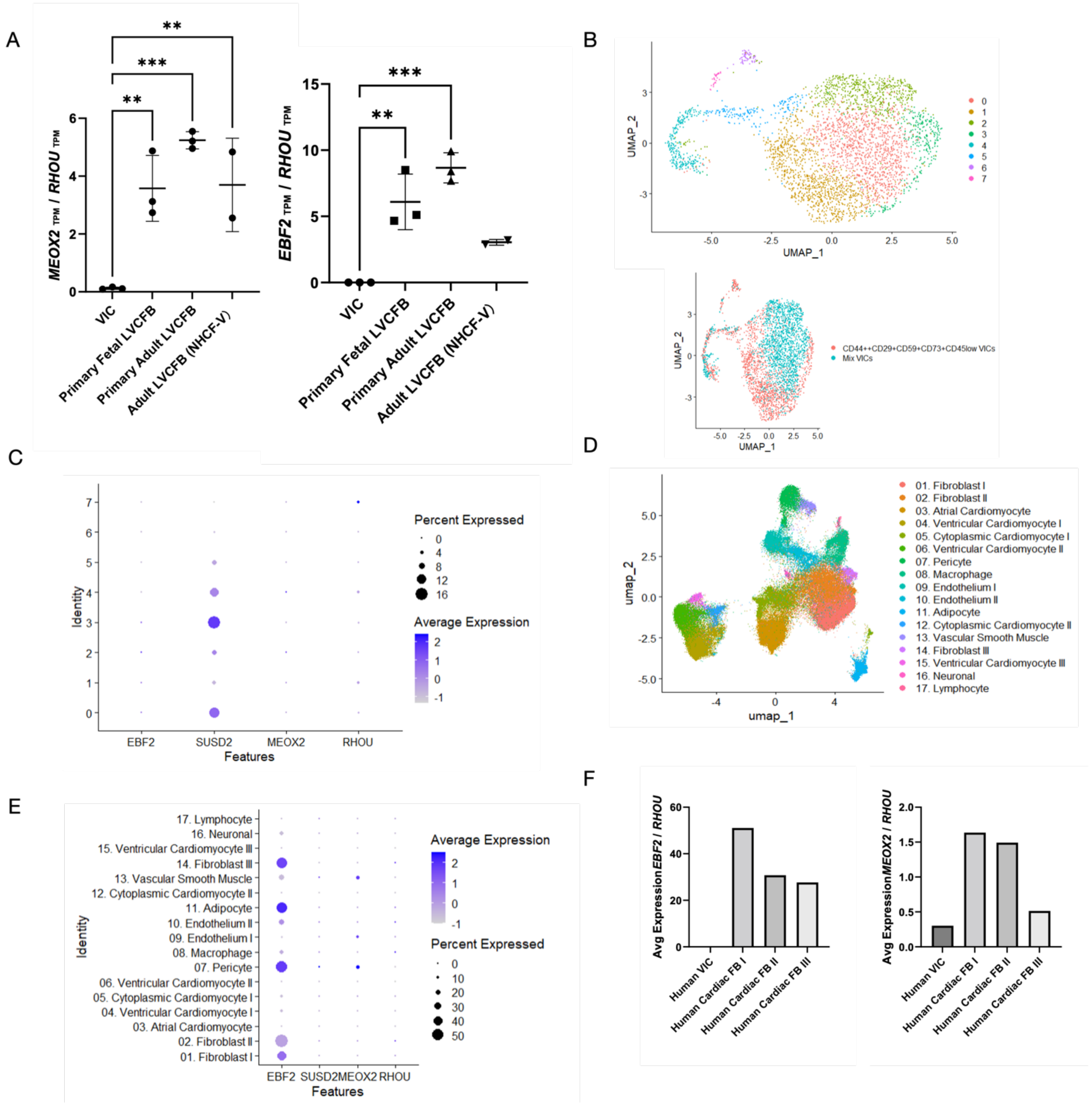

Figure S5: Marker validation using available RNA-Sequencing Datasets

Here, we remapped publicly available bulk and single cell RNA-sequencing datasets to the hg38+decoy genome to calculate TPM ratios of identified marker genes. **(A)** Bulk RNA Sequencing analysis of human primary VIC (GSE165524), human primary fetal and adult LVCFB (GSE168380), and adult LVCFBs (GSE126260). TPM ratios of *EBF2* and *MEOX2* against *RHOU* are plotted. Dots

represent biological replicates. Statistics are a one-way ANOVA followed by Dunnett's test against Human VIC sample where * is $P<0.05$, ** is $P<0.01$, and *** is $P<0.001$. **(B)** Single cell RNA-sequencing analysis of FACS-sorted human mix aortic VIC and CD44high CD29+ CD59+ CD73+ CD45low disease-driving VIC (GSE194180). UMAP demonstrating 7 VICs clusters is shown. **(C)** Dot plot demonstrating average expression and percent expression of *EBF2, MEOX2* and *RHOU*. **(D)** Single cell RNA Sequencing analysis of whole heart (SCP498, Broad Institute). UMAP demonstrating 17 different clusters is shown. Cluster 1, 2 and 14 are fibroblast clusters. **(E)** Dot plot demonstrating average expression and percent expression of *EBF2, MEOX2* and *RHOU*. **(F)** Average expression ratio of *EBF2* and *MEOX2* against *RHOU* in the two single cell RNA-sequencing datasets.

Table S1: Donor information.

**Donor**	**Gender**		**History of Hypertension**
F33	Female	33	Yes, 0-5 Years
F34	Female	34	Yes, >10 Years
M39	Male	39	Yes, Duration Unknown

Table S2: Primary and secondary antibody information.

Antibody	Vendor	Product Number	Host	Flow Cytometry (FC)	Immunostaining (ICC)	Western Blotting (WB)
Vimentin	R&D Systems	IC2105G	Pre-conjugated 488	1:100	PFA, BSA, 1:100	
CD90	BioLegend	B328102	Mouse IgG1	1:100	PFA, BSA, 1:100	
FN	Santa Cruz	Sc-8422	Mouse IgG1		PFA, BSA, 1:200	
FSP1	Millipore	ABF32	Rabbit IgG	1:500	PFA, BSA, 1:500	
TE-7	Millipore	CBL271	Mouse IgG1	1:100	PFA, BSA, 1:100	
FN EDA splice	Millipore	MAB1940	Mouse IgG1	1:200		
PDGFRα	Abcam	ab134123	Rabbit monoclonal	1:200		
COL1A1	Sigma Aldrich	C2456	Mouse IgG1		PFA, BSA, 1:100	
SUSD2	BioLegend	327401	Mouse IgG1			Milk, 1:1000
B-actin	Cell Signaling	4970S	Rabbit IgG			Milk, 1:1000
aSMA	Invitrogen	MA5-11544	Mouse IgG2a		PFA, Milk, 1:100	
EBF2	Sigma Aldrich	AV36019	Rabbit polyclonal		1:3000 (IHC)	Milk, 1:1000
EBF2	R&D Systems	AF7006	Sheep polyclonal		1:100 (IHC)	
MEOX2	Sigma Aldrich	HPA053793	Rabbit polyclonal			Milk, 1:1000
MEOX2	Thermo Fisher Scientific	H00004223	Mouse IgG1		1:500 (IHC)	Milk, 1:1000
RHOU	Thermo Fisher Scientific	PA5-69128	Rabbit polyclonal		1:1000 (IHC)	Milk, 1:1000
RHOU	Bioss	BS-1944R	Rabbit polyclonal			Milk, 1:1000
Alexa Fluor 488	Invitrogen	A11001	Goat anti-mouse IgG1	x		x
Alexa Fluor 647	Invitrogen	A21240	Goat anti-mouse IgG1	x		x
Alexa Fluor 488	Invitrogen	A21441	Chicken anti-rabbit IgG	x		x
Alexa Fluor 647	Invitrogen	A31573	Donkey anti-rabbit IgG	x		x
Alexa Fluor 647	Invitrogen	A21241	Goat anti-mouse IgG2a	x		x
IR Dye 680RD	LiCor	926-68073	Donkey anti-rabbit IgG		x	
IR Dye 800CW	LiCor	926-32212	Donkey anti-mouse IgG		x	

Hoechst	Thermo Fisher	62249				x

Table S3: Primer information

Gene	Forward	Reverse	Product length
NTRK2	ACTGCAGCGAATGACATCGG	ACAGACGCAATCACCACCAC	122
RHOU	ACATCCCTACTGCCTTCGACA	TCAGCTTGTCAAATTCATCCTGTCC	108
MEOX2	CAACTCTTCCAGCTTGGGCTC	ATTTCCTTCCTGGGAGTCTGAG	148
EBF2	CATTTTGAAGCGAGCCGCAG	TGTTGCGGATGTACCCTTGAT	188
